# Bibliometric and LDA analysis of extracellular vesicles in osteoarthritis

**DOI:** 10.1038/s41413-025-00484-3

**Published:** 2025-12-23

**Authors:** Hongyu Xie, Lin Zhao, Lunwei Kang, Weikun Meng, Jianshu Tan, Ga Liao

**Affiliations:** 1 State Key Laboratory of Oral Diseases & National Center for Stomatology & National Clinical Research Center for Oral Diseases, West China Hospital of Stomatology, Sichuan University, Chengdu, 610041 Sichuan China; 2 Orthopaedic Research Institute, Department of Orthopaedics, West China Hospital, Sichuan University, Chengdu, 610041 China; 3 State Key Laboratory of Oral Diseases & National Center for Stomatology & National Clinical Research Center for Oral Diseases & Department of Stomatology Informatics, West China Hospital of Stomatology, Sichuan University, Chengdu, 610041 Sichuan China

**Keywords:** Metabolic bone disease, Metabolic syndrome

## Abstract

Osteoarthritis (OA) is a common degenerative joint disease with complex risk factors, and its underlying mechanism remains unclear. The disease has a subtle onset and mild early symptoms, and its progression is irreversible. Current treatments do not offer a complete cure. Therefore, developing new therapies, early prevention strategies, and reliable biomarkers is essential to reduce the disease burden and improve the quality of life for OA patients. Extracellular vesicles, with their natural biocompatibility and low immunogenicity, have shown great potential in drug delivery and acellular therapies. To provide a complete understanding of the current research and future prospects of extracellular vesicles in OA, this study used bibliometric analysis and Latent Dirichlet Allocation (LDA) methods to systematically evaluate international collaborations, research hotspots, and emerging trends in the field. Our aim is to offer a scientific basis and reference for innovative OA treatment strategies and the clinical application of extracellular vesicles.

## Introduction

**Fig. 1:**
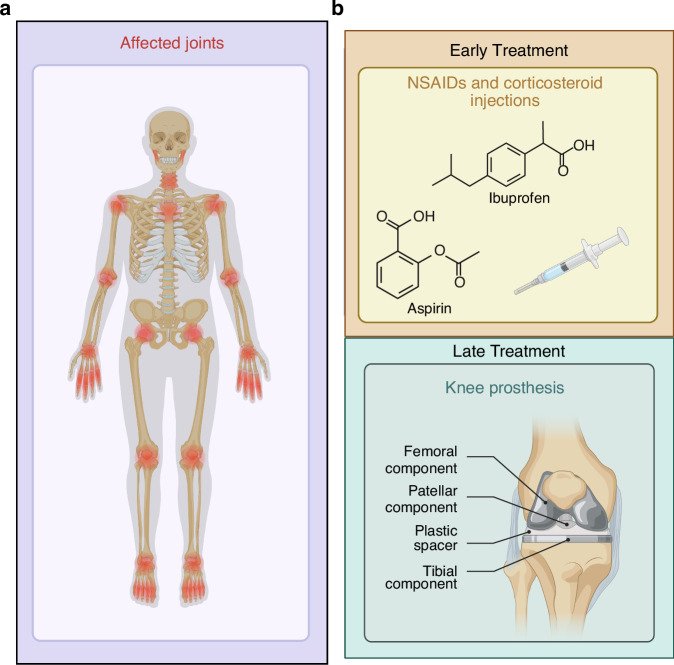
Osteoarthritis (OA) involvement and treatment continuum. (Created with BioRender.com): **a** Affected Joints: OA impacts multiple joints; **b** Treatment: Early-stage management uses NSAIDs and steroids; advanced cases may require joint replacement

**Fig. 2:**
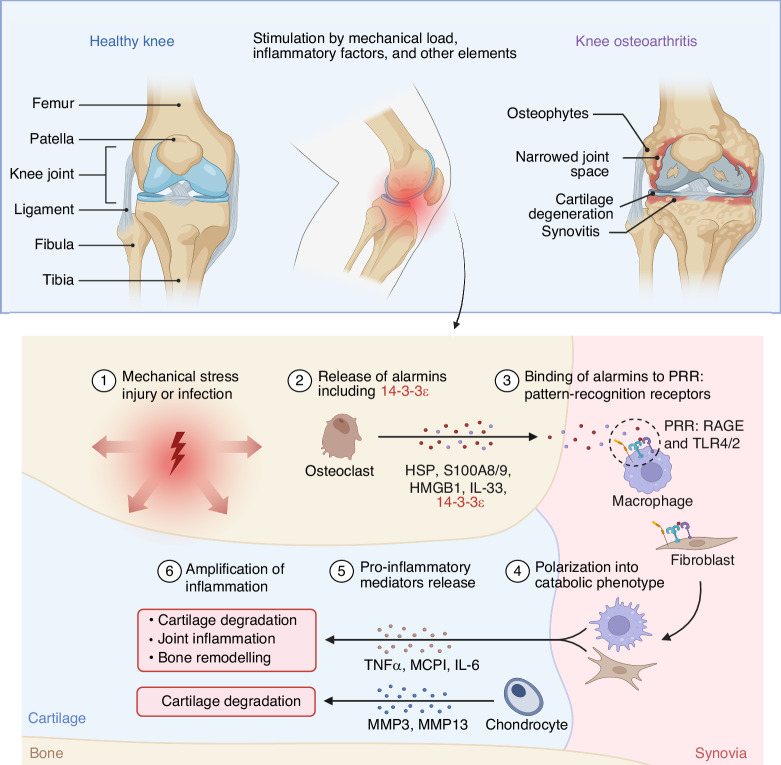
Disease Progression: Mechanical stress or infection damages cartilage, releasing alarmins that activate inflammation, leading to cartilage degradation and bone remodeling

Osteoarthritis (OA) is a highly prevalent chronic degenerative joint disorder worldwide, characterized pathologically by articular cartilage degradation, osteophyte formation, subchondral bone remodeling, and persistent low-grade inflammation in periarticular tissues.^1^ Patients typically suffer from chronic pain, stiffness, and impaired mobility, with severe cases progressing to disability, imposing substantial burdens on individual quality of life and healthcare systems (Fig. 1a). Global Burden of Disease estimates indicate that approximately 256 million people were affected by OA in 1990 (95% UI 232–282 million), rising to 595 million in 2020 (95% UI 535–656 million), with incidence continuing to increase across all age groups over the past decade; projections suggest nearly one billion individuals will be affected by 2050.^2^ Its etiology is multifactorial—driven by age, sex, obesity, joint injury, genetic predisposition, and occupational exposure—and the disease manifests marked clinical, molecular, and therapeutic heterogeneity.^3,4^ OA is better conceptualized as a spectrum of overlapping and interwoven phenotypes rather than a single uniform entity. This complexity hampers mechanistic understanding and constrains the development and generalizability of standardized intervention strategies.^5^ Current mainstream treatments focus on symptom relief (e.g., nonsteroidal anti-inflammatory drugs, intra-articular corticosteroid injections), which may transiently reduce inflammation and pain but carry risks with long-term use; for advanced disease, joint replacement can improve function, yet postoperative complications and persistent symptoms remain unresolved.^6,7^ These challenges underscore the urgent need to identify mechanistic molecular and cellular targets that could enable disease modification, slowing or reversing progression^8^ (Figs. 1b and 2).

**Fig. 3:**
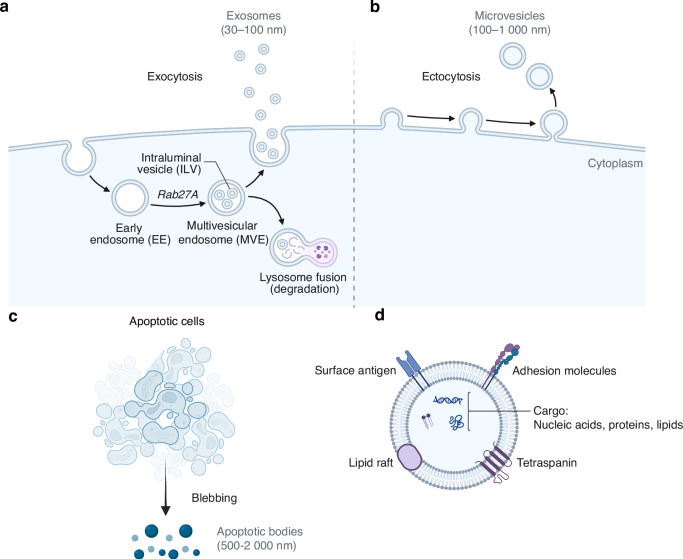
Extracellular Vesicle Biogenesis, Release Pathways, and Structure: **a** Exosome Secretion: Exosomes (30–100 nm) form in early endosomes, mature into multivesicular bodies (MVBs), and are released via Rab27A-mediated fusion; unreleased MVBs are degraded in lysosomes; **b** Microvesicle Secretion: Microvesicles (100–1 000 nm) bud directly from the plasma membrane; **c** Apoptotic Bodies: Released during apoptosis, containing cellular remnants; **d** EV Structure: EVs display surface antigens and adhesion molecules for target cell binding; their membranes include lipid rafts and tetraspanins, and they transport nucleic acids, proteins, and lipids for intercellular communication

Extracellular vesicles (EVs), as endogenous mediators of intercellular communication, have emerged as a promising acellular therapeutic modality for degenerative disorders such as OA owing to their inherent biocompatibility, low immunogenicity, and capacity to carry diverse functional cargos—including proteins, microRNAs, lipids, and other bioactive molecules.^9,10^ EVs are broadly classified by biogenesis and origin into exosomes, microvesicles, and apoptotic bodies, which differ in size, release pathways, and molecular content, yet all participate in cellular crosstalk and pathophysiological regulation^11,12^ (Fig. 3a–d). Functionally, EVs modulate inflammatory responses within the tissue microenvironment, influence cell fate decisions, and promote regenerative processes; they can also be engineered for targeted delivery of therapeutic agents. A growing body of work has elucidated specific mechanisms by which EVs affect OA pathogenesis: they facilitate the polarization of macrophages from a pro-inflammatory M1 phenotype toward a reparative M2 phenotype, suppress pro-inflammatory mediators such as IL-1β and TNF-α and downstream NF-κB signaling, thereby attenuating cartilage matrix degradation and chondrocyte apoptosis; they regulate subchondral bone metabolism by delivering miRNAs or proteins that modulate the RANKL–RANK axis, affecting the balance between osteoclast and osteoblast activity; and they enhance chondrocyte survival and matrix synthesis by transferring effectors such as miR-140, miR-223, TGF-β, and IGF-1, which upregulate SOX9 and Collagen II while inhibiting matrix metalloproteinase activity.^13–17^ These mechanistic insights have been repeatedly validated in vitro and in animal models, providing a strong biological rationale for clinical translation.

Despite abundant preclinical evidence, the clinical translation of EVs in OA remains at an early stage. For example, a randomized, triple-blind, placebo-controlled study in bilateral knee OA tested a single intra-articular injection of placental MSC-derived EVs (5 mL; 7×10^9^ particles/mL) versus saline (29 patients; 58 knees) and found no significant differences between groups for VAS, WOMAC, Lequesne scores at 2 and 6 months, or for MRI-based structural measures at 6 months, while reporting no systemic complications or severe local reactions.^18^ Taken together with broader syntheses in the field, current human data appear more consistent for safety than for efficacy, and larger, methodologically rigorous studies with longer follow-up are warranted. Key obstacles to broader implementation include heterogeneity in EV source and batch characteristics, lack of standardized isolation, quantification, and potency assays, limitations in delivery efficiency and targeting, and still-evolving regulatory pathways. Compared with conventional gene or cell-based approaches, EVs offer a complementary, acellular mechanism for information transfer and regulatory modulation; their distinctive properties and potential synergies underscore the value of systematic, literature-wide analyses—from knowledge mapping to thematic evolution—to identify critical trends and translational gaps.

Although several narrative and thematic reviews have summarized the potential of EVs in OA, the field lacks a systematic, quantitative knowledge map that reveals its evolution, international collaboration patterns, latent thematic structure, and clinical translation trajectories. Existing bibliometric and topic modeling studies often fall short in adequately justifying model settings (such as hyperparameter selection and topic number determination), ensuring transparency, controlling representational bias, and validating robustness, thereby limiting reproducibility and generalizability. Latent Dirichlet Allocation (LDA), a probabilistic generative model widely applied in scientometrics and social sciences to uncover latent thematic structures from large text corpora, requires careful attention to its assumptions, parameter tuning, and interpretability to yield reliable insights. In this study, we systematically analyzed the literature on extracellular vesicles and osteoarthritis published between January 1, 2003 and December 31, 2024, by integrating bibliometric methods with LDA topic modeling to construct a comprehensive research landscape. The objectives of this study were to map key research hotspots, elucidate global collaboration networks, model the temporal evolution of themes, and identify pathways to clinical translation, with the intent of providing rigorous, actionable guidance for subsequent mechanistic research and clinical trial planning.

## Results

### Literature growth trend

**Fig. 4:**
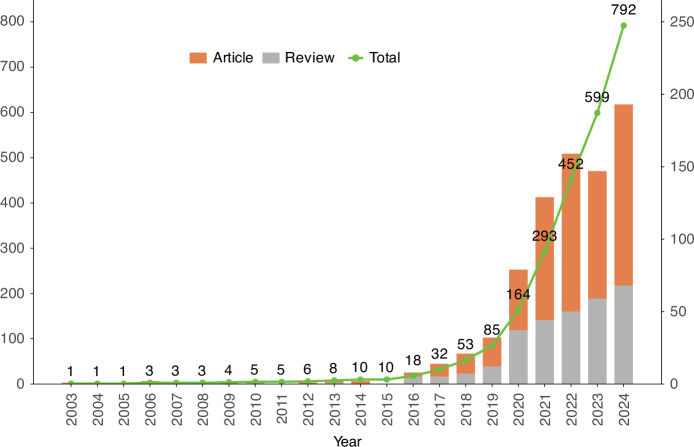
Number of annual publications related to EVs in OA research from 2003 to 2024

A total of 792 publications meeting the inclusion criteria were retained for analysis, distributed across 297 distinct journals. The field demonstrated rapid expansion, with an average annual growth rate of 28.48%, indicating increasing scientific attention to extracellular vesicles in osteoarthritis. Each article received a mean of 33.06 citations, reflecting substantive academic influence. In total, 3 958 unique authors contributed to the corpus, with six single-author papers. Collaborative behavior was prominent: the average number of co-authors per paper was 7.33, and 17.30% of publications involved international collaboration (Table 1). The annual output exhibited a clear upward trajectory, with 2017 marking the first year in which the year-over-year increase exceeded double digits. This acceleration coincides temporally with the introduction and broader adoption of improved EV isolation and characterization techniques—such as ultracentrifugation refinements, size-exclusion chromatography, and microfluidics-based platforms—as well as growing recognition of EVs’ potential as carriers of microRNAs and proteins serving as diagnostic, prognostic, and therapeutic biomarkers^19–21^ (Fig. 4).

**Table 1:** Primary Information on Research Literature Analysis

Description	Results
**MAIN INFORMATION ABOUT DATA**	
Timespan	2003-2024
Sources (Journals, Books, etc)	297
Documents	792
Annual Growth Rate %	28.48
Document Average Age	3.22
Average citations per doc	33.06
References	35 655
**DOCUMENT CONTENTS**	
Keywords Plus (ID)	1 414
Author’s Keywords (DE)	1 410
**AUTHORS**	
Authors	3 958
Authors of single-authored docs	6
**AUTHORS COLLABORATION**	
Single-authored docs	6
Co-Authors per Doc	7.33
International co-authorships %	17.30
**DOCUMENT TYPES**	
article	503
review	289

### Distribution by country/region

**Fig. 5:**
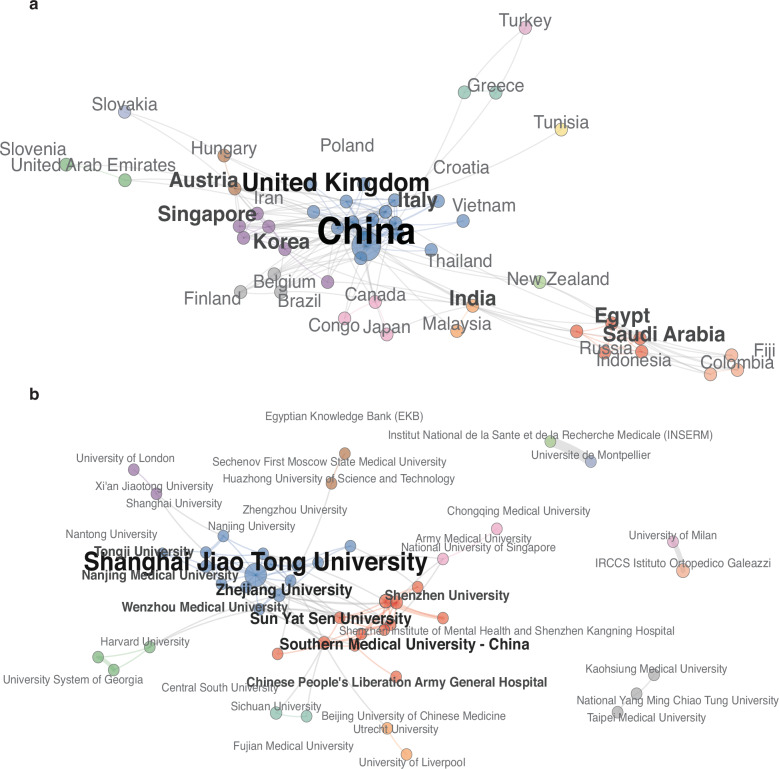
Global and Institutional Collaboration in EV–OA Research: **a** Country collaboration network (top 50): node size = publications; edge width = normalized collaboration strength; color = communities (Walktrap); layout = Fruchterman–Reingold; edges below the display threshold omitted. **b** Institution collaboration (chord diagram, top 50): sector width = output; chord thickness = number of co-authored papers; colors denote communities

The studies originated from 51 countries or regions. The top 20 contributing countries by publication count are highlighted in the collaboration network, with China (*n* = 473), the United States (*n* = 196), and Italy (*n* = 64) ranking first through third. International collaboration links among these and other countries were robust. China also led in total citations (14 436), followed by the United States (6 370) and Singapore (2 398) (Table S1). The outsized output and citation impact of China may reflect both its large population and demographic aging, which together amplify the public health urgency of osteoarthritis and spur corresponding research investment^22^ (Fig. 5a).

To assess whether high-output countries disproportionately shaped identified research hotspots and their temporal dynamics, we conducted two sensitivity analyses. In the leave-one-country-out analysis, exclusion of China, the United States, or Italy in turn resulted in high consistency for core keywords such as “cartilage,” “exosome,” “inflammation,” and “regeneration” relative to the full dataset (majority Pearson’s *r* > 0.85), indicating that these primary thematic trends are not driven solely by any single dominant country; a few lower-frequency terms (e.g., “RANKL”) showed reduced consistency when China was excluded, likely due to their sparsity and regional concentration. In the balanced bootstrap analysis, we performed 500 iterations of equal-sample resampling across China, the United States, and Italy (matching to the country with the smallest output) to construct confidence intervals for keyword occurrence under normalized contributions. Core terms (e.g., “inflammation,” “cartilage,” “exosome”) remained stable with narrow 95% confidence intervals, whereas “RANKL” displayed low and unstable prevalence, reinforcing its focal but less ubiquitous nature (Tables S2–3). In combination, the two approaches confirm that the key thematic trends are robust to the influence of any individual high-output country.

### Distribution of institutions

The 792 publications were affiliated with 2 754 distinct institutions. The top 20 institutions accounted for 353 publications (accounting for 44.57% of the total) and are summarized in Table S4. Shanghai Jiao Tong University led in publication volume (*n* = 41), followed by IRCCS Istituto Ortopedico Galeazzi (*n* = 25) and Sichuan University (*n* = 24). In terms of total citation impact, the National University of Singapore ranked highly, also achieving the highest average citations per paper among the leading institutions (Table S4). The institutional collaboration network (Fig. 5b) reveals dense inter-institutional linkages, illustrating widespread engagement in EV-related osteoarthritis research across major centers.

### Author distribution

**Fig. 6:**
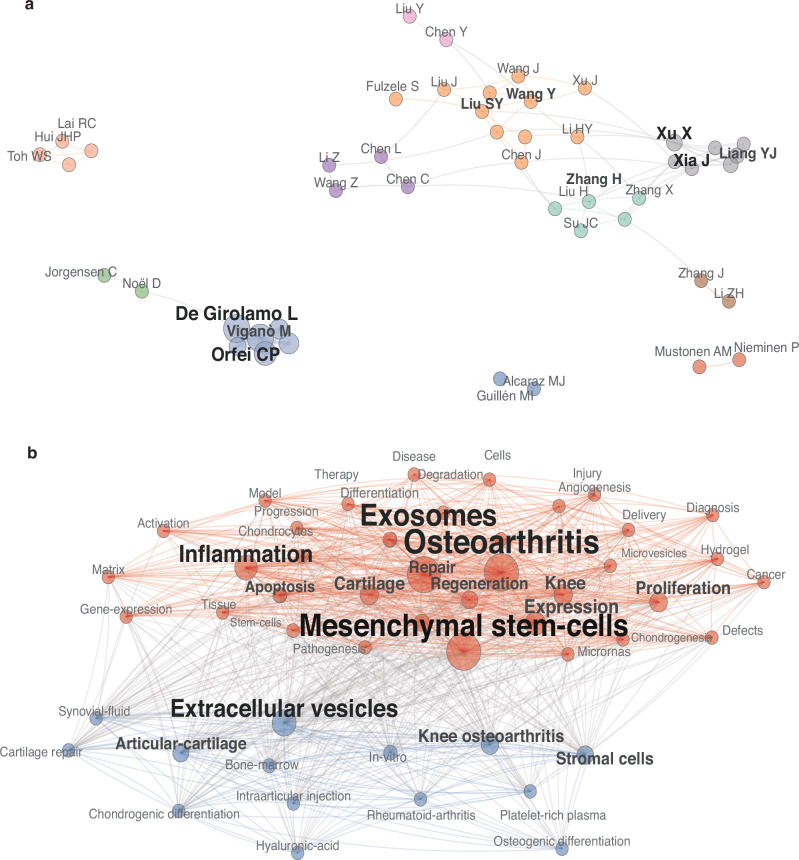
Collaboration and Keyword Networks in EV–OA: **a** Author collaboration network (top 50): node size = productivity; edges = co-authorships (≥1 shared paper); layout and community detection as in (**a**). **b** Keyword co-occurrence network: terms with ≥15 occurrences after synonym merging; node size = term frequency; edge weight = normalized co-occurrence; color = thematic clusters (Walktrap)

A total of 3 958 authors contributed to the literature. Table S5 lists the top 20 most productive authors by number of publications. DE GIROLAMO L and RAGNI E tied for the highest output (*n* = 23 each), followed by ORFEI CP (*n* = 18), COLOMBINI A (*n* = 14), and LI Y (*n* = 14). Citation-based influence metrics identify TOH WS (2 116 citations), LIM SK (2 044 citations), and LAI RC (2 029 citations) among the most highly cited researchers, suggesting their centrality in shaping the intellectual foundation of the field (Table S5). Although the co-authorship network exhibits some dispersion—partly attributable to geographic separation—many authors maintain active collaboration ties, indicating both localized and cross-regional research integration (Fig. 6a).

### Journals and citation analysis

The included studies were published in 297 journals. *The International Journal of Molecular Sciences* published the greatest number of papers (*n* = 46; total citations = 725), followed by *Frontiers in Bioengineering and Biotechnology* (*n* = 27; total citations = 727) and *Cells* (*n* = 23; total citations = 442) (Table S6). In terms of average citation impact per article, *Biomaterials*, *Theranostics*, and *Stem Cell Research & Therapy* ranked highest, indicating their role in disseminating high-impact work in this domain.

### Keyword co-occurrence

**Fig. 7:**
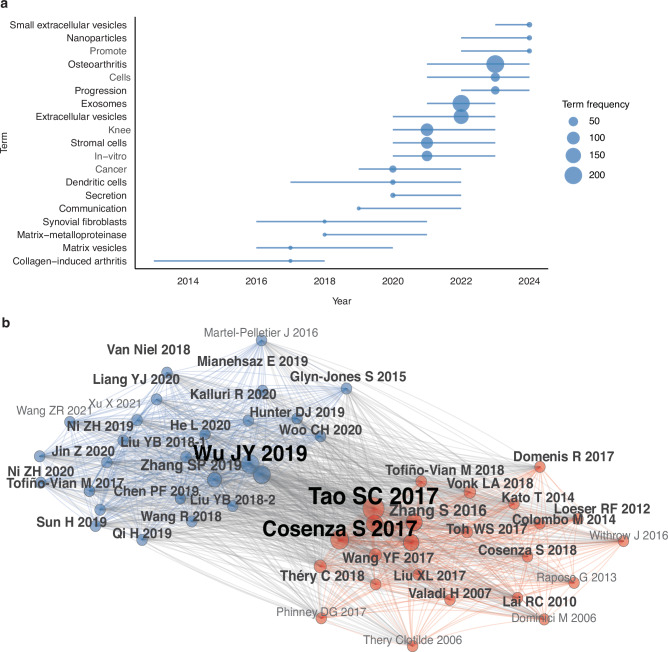
Keyword Trends and Co-citation Structure: **a** Temporal keyword trends: annual frequency trajectories for selected terms. **b** Reference co-citation network: node size = co-citation counts; edges = normalized co-citation links above threshold; colors = modular clusters (Walktrap)

After filtering terms with fewer than 15 occurrences, merging synonyms, and removing non-informative tokens, 50 keywords remained for co-occurrence analysis. Central nodes such as “mesenchymal stem cells,” “osteoarthritis,” and “exosomes” reflect tightly coupled research foci in recent years. The frequent co-occurrence of “cells” with these terms emphasizes the prominence of cell-based conceptual framing in the field. Furthermore, linkages among “inflammation,” “repair,” and “regeneration” underscore the mechanistic interest in how EVs mediate tissue healing and inflammatory modulation. Trend topic analysis demonstrated that terms including “osteoarthritis,” “mesenchymal stem cells,” and “exosomes” have substantially increased in frequency since 2021, persisting through 2023–2024, whereas earlier terms such as “matrix vesicles” and “collagen-induced arthritis” peaked around 2014–2016 and have since declined. These shifts suggest an evolving emphasis toward stem cell–derived EVs and their therapeutic roles (Figs. 6b and 7a).

### Reference citation analysis

Reference co-citation occurs when two publications (A and B) are cited together by a third publication (C). In co-citation networks, key nodes occupying central positions in the co-citation network—such as Cosenza S (2017), Zhang SP (2018), Tao SC (2017), and Wu JY (2019)—represent seminal contributions that have shaped subsequent research trajectories.^14,23–25^ For example, Tao SC’s 2017 *Theranostics* study (243 citations) demonstrated that exosomes derived from synovial mesenchymal stem cells overexpressing miR-140-5p significantly promote cartilage regeneration and prevent osteoarthritis progression in rat models, underscoring therapeutic potential.^14^ Subsequent highly cited works (e.g., Cosenza S 2017 in Scientific Reports, Zhang SP 2018 in *Biomaterials*, Wu JY 2019 in *Biomaterials*, Zhang S 2016 in *Osteoarthritis and Cartilage*, and Mao GP 2018 in *Stem Cell Research & Therapy*) expanded on mechanisms of cartilage protection, immune modulation, and tissue repair.^23–27^ The inclusion of the MISEV2018 guidelines by Théry C (2018, *Journal of Extracellular Vesicles*)—with 139 citations—highlights ongoing efforts toward methodological standardization in EV research.^28^ The overall co-citation network shows interwoven clusters, indicating strong thematic interconnectedness among different research directions in EVs and osteoarthritis (Table 2 and Fig. 7b).

**Table 2:** Top 20 most-cited references

Rank	Author	Year, journal, title	Citations	Topic	Type
1	TAO SC	2017, *Theranostics*, Exosomes derived from miR-140-5p-overexpressing human synovial mesenchymal stem cells enhance cartilage tissue regeneration and prevent osteoarthritis of the knee in a rat model	243	miR-140 Exosomes Prevent OA	Rat preclinical
2	COSENZA S	2017, *Sci. Rep.*, Mesenchymal stem cells derived exosomes and microparticles protect cartilage and bone from degradation in osteoarthritis	198	MSC EVs Treat OA	comparative study
3	ZHANG SP	2018, *Biomaterials*, MSC exosomes mediate cartilage repair by enhancing proliferation, attenuating apoptosis and modulating immune reactivity	193	MSC Exosomes Repair Cartilage	Rat defect
4	WU JY	2019, *Biomaterials*, miR-100-5p-abundant exosomes derived from infrapatellar fat pad MSCs protect articular cartilage and ameliorate gait abnormalities via inhibition of mTOR in osteoarthritis	183	miR-100 Exosomes Treat OA	DMM mouse
5	ZHANG S	2016, *Osteoarthritis Cartilage*, Exosomes derived from human embryonic mesenchymal stem cells promote osteochondral regeneration	179	MSC exosomes regenerate osteochondral tissue	Rat defect
6	MAO GP	2018, *Stem Cell Res. Ther.*, Exosomes derived from miR-92a-3p-overexpressing human mesenchymal stem cells enhance chondrogenesis and suppress cartilage degradation via targeting WNT5A	167	miR-92a Exosomes Cartilage	Preclinical exp
7	ZHANG SP	2019, *Biomaterials*, MSC exosomes alleviate temporomandibular joint osteoarthritis by attenuating inflammation and restoring matrix homeostasis	150	MSC exosomes treat TMJ-OA	Rat experimental
8	ZHU Y	2017, *Stem Cell Res. Ther.*, Comparison of exosomes secreted by induced pluripotent stem cell-derived mesenchymal stem cells and synovial membrane-derived mesenchymal stem cells for the treatment of osteoarthritis	143	iMSC vs SMMSC Exosomes	comparative study
9	THÉRY C	2018, *J. Extracell Vesicles*, Minimal information for studies of extracellular vesicles 2018 (MISEV2018): a position statement of the International Society for Extracellular Vesicles and update of the MISEV2014 guidelines	139	Minimal Information	Consensus Document
10	TOH WS	2017, *Semin. Cell Dev. Biol.*, MSC exosome as a cell-free MSC therapy for cartilage regeneration: implications for osteoarthritis treatment	135	OA treatment	Review
11	WANG YF	2017, *Stem Cell Res. Ther.*, Exosomes from embryonic mesenchymal stem cells alleviate osteoarthritis through balancing synthesis and degradation of cartilage extracellular matrix	122	ESC-MSC exosomes for OA	In vivo exp
12	HUNTER DJ	2019, *Lancet*, Osteoarthritis	115	Pathogenesis & Management	Clinical Seminar
13	VONK LA	2018, *Theranostics*, Mesenchymal stromal/stem cell-derived extracellular vesicles promote human cartilage regeneration in vitro	112	MSC-EV cartilage repair	In vitro regen
14	HE L	2020, *Stem Cell Res. Ther.*, Bone marrow mesenchymal stem cell-derived exosomes protect cartilage damage and relieve knee osteoarthritis pain in a rat model of osteoarthritis	103	BMSC exosomes in OA	In vitro
15	KALLURI R	2020, *Science*, The biology, function, and biomedical applications of exosomes	102	Exosome biology & applications	Review
16	LIU YB	2018, *Cell Cycle*, MSC-derived exosomes promote proliferation and inhibit apoptosis of chondrocytes via lncRNA-KLF3-AS1/miR-206/GIT1 axis in osteoarthritis	101	MSC exosomes boost chondrogenesis	In vivo mech
17	KATO T	2014, *Arthritis Res. Ther.*, Exosomes from IL-1β stimulated synovial fibroblasts induce osteoarthritic changes in articular chondrocytes	92	MSC exosomes in OA	Mechanistic study
18	TOFIÑO-VIAN M	2018, *Cell Physiol. Biochem.*, Microvesicles from human adipose tissue-derived mesenchymal stem cells as a new protective strategy in osteoarthritic chondrocytes	90	AD-MSC EV Chondroprotection	In vitro/ex vivo
19	LIU YB	2018, *Biochem. J.*, Exosomal KLF3-AS1 from hMSCs promoted cartilage repair and chondrocyte proliferation in osteoarthritis	88	Exosomal lncRNA, Cartilage Repair	Preclinical cell
20	NI ZH	2020, *Bone Res.*, Exosomes: roles and therapeutic potential in osteoarthritis.	80	3D Exosomes therapeutic potential in OA	Review

### LDA-based topic modeling

We applied LDA to the corpus (excluding any records lacking abstracts) to identify 15 dominant latent topics. Topic labels were assigned after manual review of the top 20 terms and representative documents for each topic. Prominent topics included “Topic 6: EV Regenerative Medicine” (*n* = 80; 10.10%) and “Topic 8: Intercellular Communication” (*n* = 78; 9.85%), followed by “Topic 1: IL-1β-induced Proliferation” (*n* = 70; 8.84%), “Topic 7: OA Treatment” (*n* = 70; 8.84%), and “Topic 4: Chondrogenic Differentiation” (*n* = 68; 8.59%) (Table 3).

**Table 3:** Topics discovered from 792 articles published between 2003 and 2024

Topic	Prevalence	Top Terms	Label	N
Topic 1	0.08	il-1_beta, il-1_beta-induced, proliferation_migration, mesenchymal_stem, osteoarthritis_oa, western_blot, chondrocyte_proliferation, cell_viability, flow_cytometry, oa_model	IL-1β-induced Proliferation	70
Topic 2	0.06	extracellular_vesicles, vesicles_evs, osteoarthritis_oa, synovial_fluid, micrornas_mirnas, stem_cells, joint_disease, gene_expression, surface_markers, evs_derived	Synovial Fluid EVs	47
Topic 3	0.06	stem_cells, mesenchymal_stem, cells_mscs, msc-derived_exosomes, cartilage_regeneration, tissue_regeneration, osteoarthritis_oa, articular_cartilage, derived_mscs, growth_factors	MSC Exosome Regeneration	54
Topic 4	0.08	stem_cells, cartilage_regeneration, mesenchymal_stem, stem_cell, chondrogenic_differentiation, cartilage_repair, bone_marrow, extracellular_vesicles, vitro_vivo, osteoarthritis_oa	Chondrogenic Differentiation	68
Topic 5	0.06	cartilage_regeneration, stem_cells, cartilage_repair, articular_cartilage, stem_cell, extracellular_vesicles, tissue_engineering, regenerative_medicine, mesenchymal_stem, drug_delivery	Cartilage Tissue Engineering	40
Topic 6	0.10	extracellular_vesicles, vesicles_evs, mesenchymal_stem, cells_mscs, stem_cells, regenerative_medicine, tissue_repair, msc-derived_evs, stromal_cells, stem_cell	EV Regenerative Medicine	80
Topic 7	0.09	osteoarthritis_oa, treatment_oa, oa_treatment, oa_progression, oa_common, joint_disease, degenerative_joint, pathogenesis_oa, articular_cartilage, progression_oa	OA Treatment	70
Topic 8	0.10	intercellular_communication, stem_cells, rheumatoid_arthritis, joint_diseases, inflammatory_diseases, bone_diseases, mesenchymal_stem, nucleic_acids, diseases_including, osteoarthritis_oa	Intercellular Communication	78
Topic 9	0.07	osteoarthritis_oa, intra-articular_injection, stem_cells, oa_mice, oa_model, oa_progression, treatment_oa, articular_cartilage, mesenchymal_stem, cartilage_degeneration	Intra-articular Injection	59
Topic 10	0.05	exosomes_derived, stem_cells, mesenchymal_stem, extracellular_vesicle, proliferation_migration, articular_cartilage, cartilage_repair, extracellular_matrix, study_aimed, articular_chondrocytes	Exosome-mediated Repair	34
Topic 11	0.07	il-1_beta, extracellular_vesicles, nanoparticle_tracking, oa_chondrocytes, tracking_analysis, osteoarthritis_oa, gene_expression, vesicles_evs, electron_microscopy, transmission_electron	Nanoparticle Tracking	48
Topic 12	0.04	stem_cells, mesenchymal_stem, subchondral_bone, tmj_oa, infrapatellar_fat, exosomes_derived, fat_pad, temporomandibular_joint, articular_cartilage, joint_osteoarthritis	TMJ Osteoarthritis	27
Topic 13	0.03	knee_oa, msc_exosomes, cartilage_repair, safety_efficacy, hyaluronic_acid, extracellular_vesicles, osteoarthritis_oa, knee_osteoarthritis, growth_factors, wharton’s_jelly	Knee OA Exosomes	29
Topic 14	0.05	synovial_fluid, differentially_expressed, rheumatoid_arthritis, synovial_fibroblasts, arthritis_ra, oa_patients, synovial_fluid-derived, mass_spectrometry, patients_ra, osteoarthritis_oa	Synovial Fluid Proteomics	40
Topic 15	0.06	extracellular_vesicles, stromal_cells, osteoarthritis_oa, mesenchymal_stromal, vesicles_evs, cells_mscs, cellular_senescence, senescent_cells, secretory_phenotype, promising_therapeutic	Senescent EVs	48

**Fig. 8:**
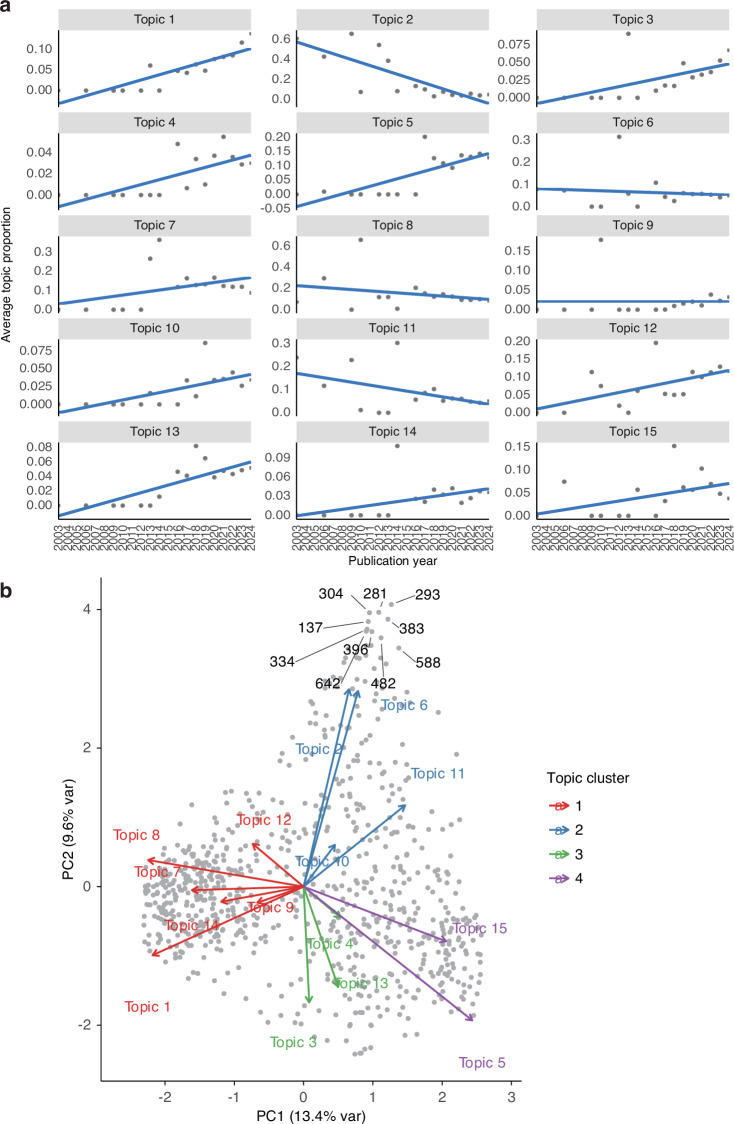
Topic Dynamics and Document–Topic Structure: **a** Temporal trends of latent topics in extracellular vesicle research on osteoarthritis (2003–2024). Annual mean posterior proportions are shown for 15 topics (Topic 1–Topic 15), with per-year observations (dots) and fitted linear trends. The slope direction and magnitude summarize rising versus declining thematic attention over time. **b** Husson–Jongmans biplot of the document–topic distribution. Principal component analysis of the topic posterior matrix (θ). PC1 and PC2 explain 10.3% and 8.6% of the variance, respectively. Gray points represent individual documents; colored arrows represent topic vectors (arrow length indicates contribution to the component; direction indicates association with the axes). Topics form four clusters (cluster 1–4; colors as in legend), highlighting thematic grouping and document–topic relationships

To examine temporal dynamics, linear regression models were fitted for each topic, with publication year as the independent variable and annual mean posterior topic probability as the dependent variable. Trends were classified based on the slope and statistical significance (threshold: *P* < 0.01). Topics showing significant positive trends included Topic 1 (IL-1β-induced proliferation; slope = 0.005 8, *P* = 0.004 7), Topic 4 (osteogenic/chondrogenic differentiation; slope = 0.005 4, *P* < 0.000 1), Topic 5 (cartilage tissue engineering; slope = 0.004 0, *P* < 0.000 1), Topic 7 (OA therapeutic strategies; slope = 0.006 3, *P* = 0.000 3), and Topic 9 (intra-articular delivery; slope = 0.004 4, *P* = 0.002 2), indicating their rising prominence. In contrast, Topic 14 (synovial proteomics; slope = –0.032 9, *P* = 0.001 9) showed a significant decline, while the remaining topics exhibited no significant temporal change (*P* > 0.01), suggesting relative stability (Fig. 8a). These upward and downward trends were further highlighted in a volcano-style plot mapping slope versus –log_10_*P* (Fig. S1).

Principal component analysis of the document-topic distribution matrix θ was visualized via a Husson–Jongmans biplot. The first two principal components explained approximately 13.4% (PC1) and 9.6% (PC2) of the variance. In the biplot gray points represent individual documents, and colored arrows denote topic vectors: longer arrows indicate stronger contributions to the component, and documents located in the direction of an arrow have higher posterior probabilities for that topic. Four major clusters emerged: Cluster 1 (right side) encompassed inflammatory model–related research such as IL-1β-induced proliferation, differentiation, and intra-articular delivery; Cluster 2 (upper region) focused on synovial EV characterization and proteomics; Cluster 3 (lower-left quadrant) included MSC-derived EV regeneration, cartilage tissue engineering, and therapeutic strategy development; and Cluster 4 (negative PC1 axis) emphasized foundational mechanisms like intercellular communication and EV regenerative biology (Fig. 8b).

## Discussion

This study combined bibliometric mapping with LDA to depict the knowledge landscape, thematic evolution, and international collaboration patterns of EVs research in OA from 2003 to 2024. Overall, the field appears to be expanding from single-target explorations toward an integrated path linking inflammatory regulation, osteochondral unit remodeling, engineering-enabled delivery, and quantitative evaluation. China, the United States, and Italy emerge as major collaboration hubs. These thematic shifts are directionally aligned with clinical aims—pain relief, functional improvement, and structural preservation—suggesting that future work may increasingly emphasize clinical verifiability and translational relevance; however, the present evidence is insufficient to assert a definitive stage transition.

Mechanistically, LDA-identified rising topics are consistent with established biology along three interconnected axes. First, the immune–inflammatory axis: EV cargo (miRNAs and proteins) is associated with dampening of NF-κB signaling, reduced IL-1β and TNF-α activity, and promotion of macrophage polarization from M1 to reparative M2 phenotypes; emerging data also suggest modulation of autophagy and metabolic stress via PI3K–AKT–mTOR/AMPK pathways, collectively mitigating inflammation-driven matrix degradation.^29,30^ Second, the cartilage/ECM homeostasis axis: increasing attention to cartilage repair aligns with EV-mediated delivery of protective factors (e.g., regulators of SOX9 and type II collagen), inhibition of MMP/ADAMTS, and pathway cross-talk through TGF-β/Smad and Wnt/β-catenin that helps maintain chondrocyte phenotype and matrix balance.^31,32^ Third, the bone metabolism/subchondral bone axis: regulation around the RANKL–RANK–OPG triad has gained traction, with EVs potentially transmitting signals that influence osteoclast–osteoblast coupling and the subchondral microenvironment, thereby indirectly affecting pain and joint mechanics.^33,34^ Importantly, whether such mechanisms translate into consistent benefit at the population level depends on the coupling of delivery, retention, and exposure; inadequate spatial targeting or exposure duration can dilute true effects. Against this backdrop, engineering-enabled delivery and evaluation frameworks are pivotal for bridging experimental effects and clinically detectable outcomes. Hydrogels, targeting peptides, photo-crosslinkable/phototriggered systems, and surface engineering have extended intra-articular residence, enhanced tissue localization, and prolonged pharmacodynamic activity in preclinical settings.^35–37^ In practice, aligning critical quality attributes (e.g., size, purity, stability/shelf-life, functional cargo) with potency assays (standardized in vitro functional readouts) within a coherent quality management loop may improve cross-center comparability and batch release consistency. Where feasible, adopting a multidimensional endpoint framework—pain/function scales (e.g., VAS, WOMAC), quantitative imaging (e.g., MRI T2/T2* mapping, cartilage thickness or voxel-based metrics, ultrasound-based measures), and synovial fluid/serum biomarkers (e.g., IL-1β, TNF-α, CTX-II, COMP)—with sufficiently long follow-up can help capture delayed structural and functional effects.^38,39^

Clinical exploration of intra-articular EV/MSC products remains at an early stage, with findings to date focusing more on safety and tolerability than on consistent efficacy. A multicenter Phase I open-label study using cell-derived acellular conditioned medium (CCM) in knee OA (*n* = 12) primarily assessed injection tolerability and adverse events without reporting efficacy endpoints.^40^ A triple-blind, placebo-controlled Phase II trial (*n* = 29, bilateral-knee randomization) of placental MSC-derived EVs indicated overall safety but no significant differences in VAS, WOMAC, or MRI outcomes.^18^ Preclinically, peptide-modified, photo-polymerizable hydrogel–embedded exosomes improved joint localization and biological effects in animal and in vitro models,^35^ yet human validation is pending. Broader reviews have summarized regenerative potential and relatively low immunogenicity of MSC/EV therapeutics while highlighting heterogeneity in dose expression, preparation, routes of administration, and follow-up duration.^41,42^ Taken together, current human data are more consistent for safety than for efficacy, likely reflecting limited sample sizes, variability in blinding and controls, uncertainty in dose/frequency, differences in delivery strategies, and short follow-up.

In light of these patterns, several directions may be considered for subsequent work. First, explore prespecified phenotype-based stratification (e.g., pain-dominant, synovitis-dominant, structurally progressive OA) to reduce heterogeneity and improve power to detect true effects. Second, harmonize dose expression (particle number and/or total protein) and systematically evaluate exposure–response relationships in conjunction with delivery strategies. Third, where conditions permit, prioritize multicenter, randomized, double-blind designs and include engineering-enabled delivery as a prespecified comparative strategy. Fourth, refine a multidimensional endpoint framework and extend follow-up (e.g., 12–24 months) to better align symptom, function, structure, and biomarker trajectories. Cross-disciplinary collaboration—materials science, microfluidics, imaging, and rheumatology/orthopedics—together with shared data and sample infrastructures, may shorten the cycle from discovery to validation to application.

Strengths. (1) Integration of bibliometrics with LDA provides a coherent view across temporal trends, collaboration networks, and latent topics, and relates these to biological mechanisms; (2) the mapping of rising topics to engineering delivery and evaluation highlights leverage points along the translational chain and informs prioritization; (3) the narrative links mechanistic signals with clinically testable elements, enabling a continuous line from hotspot identification to plausible application scenarios. Scope and methodological premises. This analysis relies primarily on WoSCC and English-language literature, which may underrepresent other languages and gray literature. LDA is based on the bag-of-words assumption, and topic naming/aggregation involves some human judgment. Bibliometrics and topic modeling are intended to provide a navigation map of the research landscape and its evolution; they do not constitute causal inference. These premises are common to studies of this type and should be interpreted alongside independent experimental and clinical evidence to arrive at robust conclusions.

## Conclusion

This study integrates bibliometric mapping and LDA topic modeling to delineate the evolving landscape of EVs research in OA. Emerging themes converge along three interconnected axes—inflammatory regulation, cartilage/ECM homeostasis, and bone metabolism/subchondral remodeling—together with increasing emphasis on engineering-enabled delivery and quantitative evaluation. In human studies, evidence for intra-articular EV/MSC products is more consistent for safety than for efficacy, with the latter likely affected by heterogeneity in study design, dosing and frequency, delivery strategies, endpoints, and follow-up duration. Grounded in a structured, data-driven synthesis, the present work provides clear trend insights and translational leverage points to guide prioritization and optimization of mechanistic and clinical evaluations; interpretation alongside independent experimental and clinical data will further enhance robustness. Looking ahead, harmonizing dose expression and its linkage to critical quality attributes/potency assays, systematically evaluating exposure–response in conjunction with delivery strategies, incorporating phenotype-based stratification within multicenter randomized, double-blind frameworks, and employing multidimensional endpoints with adequate follow-up may better define the clinical value of EV-based interventions in OA.

## Materials and methods

### Search strategy

Data for this study were sourced from Clarivate Web of Science Core Collection (WoSCC), one of the most widely used databases for scientific literature. WoSCC comprises the Science Citation Index Expanded (SCI-Expanded), Emerging Sources Citation Index (ESCI), Conference Proceedings Citation Index—Science (CPCI-S), and Conference Proceedings Citation Index—Social Sciences & Humanities (CPCI-SSH). The search query employed was as follows: TS = (exosome* OR exosomes OR exosomal OR “extracellular vesicle” OR “extracellular vesicles” OR “extracellular particle” OR “extracellular particles” OR “microvesicle” OR “microvesicles” OR “Shedding Microvesicle” OR “Shedding Microvesicles” OR “Secretory Vesicle” OR “Secretory Vesicles” OR “Cell-Derived Microparticle” OR “Cell-Derived Microparticles” OR “Apoptotic Bodies” OR “Apoptotic Blebs” OR “Apoptotic Vesicles” OR “Cell Fragments” OR “ApoBDs”) AND TS = (Osteoarthritides OR Osteoarthrosis OR Osteoarthroses OR Arthritis, Degenerative OR Arthritides, Degenerative OR Degenerative Arthritides OR Degenerative Arthritis OR Arthrosis OR Arthroses OR Osteoarthrosis Deformans OR osteoarthritis).

The time span was restricted to publications between January 1, 2003 and December 31, 2024. Only articles and reviews published in English were retained; early access and other non-final publication types were excluded. The initial retrieval yielded 886 records. All records were exported in WoS plaintext format and imported into R (version 4.2.3) for deduplication and screening using the packages bibliometrix, dplyr, stringr, and readr. Deduplication proceeded in two stages: first, records were deduplicated based on DOI (DI) by retaining the earliest indexed UT for duplicated DOIs; second, for records without DOI, titles, first authors, and publication years were normalized (e.g., case folding, whitespace trimming) and deduplicated, resulting in 877 unique documents. Thereafter, rule-based metadata and keyword text mining were applied to classify each document into “included,” “excluded,” or “uncertain” categories. Classification criteria required concurrent evidence of osteoarthritis-related terms (e.g., “osteoarthritis,” “degenerative joint disease”) and extracellular vesicle–related terms (e.g., “extracellular vesicle,” “exosome”) in the title/abstract/keywords, and consistency with the pre-specified document type, language, and retraction status. To validate the automated classification, we performed manual checks: for both the “included” and “excluded” sets, 15% of each were randomly sampled and independently reviewed, yielding a disagreement rate below 5%. All “uncertain” records were manually adjudicated. The final corpus comprised 792 articles and reviews, which were retained for subsequent bibliometric and topic modeling analyses. All cleaned data were stored in UTF-8 plain text and CSV formats. Downstream analyses were conducted in R (including bibliometrix v4.0.0 and supporting packages) and Microsoft Excel 2016. As all data were publicly available bibliographic records, no ethical approval was required (Fig. S2).

### Bibliometric analysis

Bibliometric analysis quantifies characteristics of the scientific literature to map knowledge structures and dynamics. For the 792 included documents, we conducted a systematic bibliometric assessment in R using bibliometrix (v4.0.0) and its biblioshiny interface.^43^ First, annual publication counts were computed using biblioAnalysis() and visualized to depict temporal trends from 2003 to 2024. Collaboration and co-occurrence networks were constructed for countries/regions, institutions, authors, keywords, and cited references. For each network, only the top 50 nodes by publication volume or co-occurrence frequency were retained to focus on the most influential entities. Network layouts were generated primarily using the Fruchterman–Reingold force-directed algorithm to facilitate interpretability; a circular layout was also inspected for robustness in presentation. Association strength normalization was applied to edge weighting to control for size effects, and community detection was performed using the Walktrap algorithm. Isolated nodes were removed (minimum edge weight = 1) to enhance clarity. Networks were reproduced using igraph and visualized with ggraph, with node size and edge width scaled to reflect collaboration intensity and information flow magnitude. Temporal keyword prominence was assessed by calculating annual frequencies and identifying emerging terms whose occurrence exceeded the historical mean plus two standard deviations for that year, thereby defining “burst” periods of keyword prominence. Co-citation and cited reference networks employed analogous construction strategies to reveal foundational literature in the field. To evaluate the influence of geographic concentration on hotspot identification and thematic evolution, two complementary sensitivity analyses were conducted. First, a leave-one-country-out analysis was implemented: in turn, publications from China, the United States, or Italy were excluded, and the annual frequency trajectories of core keywords (e.g., “cartilage,” “exosome,” “inflammation,” “regeneration”) were recalculated; Pearson correlation coefficients between each reduced set and the full sample were computed to assess trend consistency. Second, equalized bootstrap sampling was performed among China, the United States, and Italy: for each of 500 iterations, an equal number of documents (matched to the country with the smallest output) were randomly sampled from the three countries to construct balanced keyword occurrence distributions, from which confidence intervals were derived to quantify robustness against output volume bias.

### Topic modeling

To uncover latent thematic structures in the corpus, we applied LDA, a hierarchical Bayesian extension of probabilistic latent semantic analysis (PLSA), capable of discovering underlying topics from large text corpora.^44^ Text preprocessing was performed with the quanteda package on titles, abstracts, and author keywords: all text was converted to lowercase; punctuation, numbers, and English stopwords were removed; and bigrams were generated to capture multi-word expressions. A document-term matrix (DTM) was constructed, retaining terms appearing in at least three documents and discarding any resulting empty documents.

LDA modeling was executed using topicmodels::LDA(…, method = “Gibbs”). A grid search over model hyperparameters was conducted with topic number k ∈ {5,10,15,20}, Dirichlet priors α ∈ {0.01,0.05,0.1}, and β ∈ {0.005,0.01,0.05}. Model selection was guided jointly by perplexity and topic coherence metrics; the optimal configuration was chosen as k = 15, α = 0.01, and β = 0.05. Each selected model was trained with Gibbs sampling for 1 000 iterations, including a burn-in period of 500 iterations. Topic stability was assessed via 20 bootstrap resamplings, computing the average cosine similarity of topic-word distributions across resamples to quantify reproducibility. The top 20 high-weight terms for each topic were manually reviewed and used to assign interpretable topic labels. To characterize temporal evolution of identified topics, annual average posterior topic probabilities were computed. For each topic, linear regression was performed with year as the independent variable and mean topic probability as the dependent variable; the significance and directionality of the slope were used to infer rising or declining thematic trends. To explore multidimensional relationships between documents and topics, the document-topic distribution matrix θ was subjected to principal component analysis (PCA), and a Husson–Jongmans biplot (HJ-Biplot) was generated to jointly display document scores and topic vectors in two-dimensional principal component space, illuminating inter-topic associations and representative documents.

## Supplementary information

Table S1-S6

Figure S2

Figure S1

Supplementary Figures Legends

## Data Availability

The bibliographic source records analyzed in this study were retrieved from the Clarivate Web of Science Core Collection (WoSCC) using the search strategy and date range detailed in the Methods. Because WoSCC content is subject to license restrictions, the raw records cannot be redistributed by the authors. Researchers with institutional access can reproduce the dataset by executing the stated query over the stated time window in WoSCC.
