# Supplementary material for: Bibliometric and LDA analysis of extracellular vesicles in osteoarthritis
Source: Bone Res. 2025 Dec 23;13:105. doi: 10.1038/s41413-025-00484-3 (PMC12722249; doi:10.1038/s41413-025-00484-3)
Supplement: Supplementary file 1 — Table S1-S6 [file 41413_2025_484_MOESM1_ESM.docx]

Table S1 Characteristics of the top 20 countries/Regions with the most publications

| **Rank** | **Country** | **Publications** | **Citations (rank)** | **Average citations (rank)** | **Betweenness centrality** |
| --- | --- | --- | --- | --- | --- |
| 1 | China | 443 | 14436(1) | 32.59(10) | 0.19 |
| 2 | United States | 196 | 6370(2) | 32.50(11) | 0.22 |
| 3 | Italy | 64 | 1623(4) | 25.36(15) | 0.09 |
| 4 | South Korea | 38 | 1007(6) | 26.50(14) | 0.02 |
| 5 | France | 21 | 1116(5) | 53.14(2) | 0.04 |
| 6 | Spain | 21 | 667(9) | 31.76(12) | 0.02 |
| 7 | Germany | 20 | 686(7) | 34.30(9) | 0.21 |
| 8 | Iran | 19 | 465(12) | 24.47(16) | 0.00 |
| 9 | Australia | 18 | 671(8) | 37.28(5) | 0.01 |
| 10 | Taiwan | 18 | 373(14) | 20.72(18) | 0.01 |
| 11 | Netherlands | 16 | 641(10) | 40.06(4) | 0.10 |
| 12 | India | 15 | 212(18) | 14.13(20) | 0.12 |
| 13 | Singapore | 14 | 2398(3) | 171.29(1) | 0.00 |
| 14 | Japan | 12 | 441(13) | 36.75(6) | 0.00 |
| 15 | Switzerland | 12 | 521(11) | 43.42(3) | 0.01 |
| 16 | Austria | 10 | 154(20) | 15.40(19) | 0.09 |
| 17 | Canada | 10 | 297(16) | 29.70(13) | 0.03 |
| 18 | Ireland | 9 | 329(15) | 36.56(7) | 0.00 |
| 19 | Finland | 8 | 183(19) | 22.88(17) | 0.00 |
| 20 | Hungary | 7 | 249(17) | 35.57(8) | 0.01 |

Table S2 Stability of core keyword trends after excluding major countries

| **Term** | **no China** | **no USA** | **no Italy** |
| --- | --- | --- | --- |
| cartilage | 0.990 | 0.867 | 0.999 |
| exosome | 0.953 | 0.816 | 0.991 |
| inflammation | 0.979 | 0.898 | 0.992 |
| mir | 0.947 | 0.600 | 0.997 |
| osteoblast | 0.877 | 0.994 | 0.996 |
| osteoclast | 0.783 | 0.996 | 0.994 |
| rankl | 0.480 | 1.000 | 1.000 |
| regeneration | 0.966 | 0.873 | 0.999 |

Table S3 Balanced bootstrap estimates of keyword prevalence

| **Term** | **Median Proportion** | **95% CI Lower** | **95% CI Upper** |
| --- | --- | --- | --- |
| cartilage | 0.572 | 0.503 | 0.651 |
| exosome | 0.484 | 0.409 | 0.553 |
| inflammation | 0.352 | 0.277 | 0.425 |
| mir | 0.358 | 0.283 | 0.428 |
| regeneration | 0.208 | 0.151 | 0.274 |
| osteoblast | 0.019 | 0.000 | 0.044 |
| osteoclast | 0.019 | 0.000 | 0.044 |
| rankl | 0.000 | 0.000 | 0.0063 |

Table S4 Characteristics of the top 20 institutions based on publications

| **Rank** | **Institutions** | **Country** | **Publications** | **Citations (rank)** | **Average citations (rank)** | **Betweenness centrality** |
| --- | --- | --- | --- | --- | --- | --- |
| 1 | SHANGHAI JIAO TONG UNIV | CHINA | 41 | 1825(2) | 44.51(9) | 0.07 |
| 2 | IRCCS IST ORTOPED GALEAZZI | ITALY | 25 | 532(11) | 21.28(16) | 0.02 |
| 3 | SICHUAN UNIV | CHINA | 24 | 637(10) | 26.54(13) | 0.01 |
| 4 | ZHEJIANG UNIV | CHINA | 21 | 1251(3) | 59.57(3) | 0.07 |
| 5 | CHINESE UNIV HONG KONG | CHINA | 19 | 1112(5) | 58.53(4) | 0.06 |
| 6 | SUN YAT SEN UNIV | CHINA | 19 | 1234(4) | 64.95(2) | 0.06 |
| 7 | TONGJI UNIV | CHINA | 18 | 376(14) | 20.89(17) | 0.03 |
| 8 | FUDAN UNIV | CHINA | 17 | 437(12) | 25.71(14) | 0.01 |
| 9 | HUAZHONG UNIV SCI & TECHNOL | CHINA | 17 | 322(17) | 18.94(18) | 0.04 |
| 10 | NANJING MED UNIV | CHINA | 17 | 809(7) | 47.59(8) | 0.01 |
| 11 | SHENZHEN UNIV | CHINA | 17 | 913(6) | 53.71(6) | 0.02 |
| 12 | CENT SOUTH UNIV | CHINA | 16 | 437(13) | 27.31(12) | 0.01 |
| 13 | SOUTHERN MED UNIV | CHINA | 16 | 347(15) | 21.69(15) | 0.05 |
| 14 | NATL UNIV SINGAPORE | SINGAPORE | 14 | 2398(1) | 171.29(1) | 0.05 |
| 15 | CHINA MED UNIV | CHINA | 13 | 645(9) | 49.62(7) | 0.01 |
| 16 | ZHENGZHOU UNIV | CHINA | 13 | 201(19) | 15.46(20) | 0.03 |
| 17 | ANHUI MED UNIV | CHINA | 12 | 659(8) | 54.92(5) | 0.01 |
| 18 | XI AN JIAO TONG UNIV | CHINA | 12 | 107(21) | 8.92(21) | 0.01 |
| 19 | CHINESE PEOPLES LIBERAT ARMY GEN HOSP | CHINA | 11 | 339(16) | 30.82(10) | 0.00 |
| 20 | NAVAL MED UNIV | CHINA | 11 | 307(18) | 27.91(11) | 0.02 |

Table S5 Characteristics of the top 20 authors based on publications and citations

| **Rank** | **Most productive authors (rank by number)** | **Publications** | **Citations** | **Most productive authors (rank by citation)** | **Publications** | **Citations** |
| --- | --- | --- | --- | --- | --- | --- |
| 1 | DE GIROLAMO L | 23 | 470 | TOH WS | 11 | 2116 |
| 2 | RAGNI E | 23 | 503 | LIM SK | 8 | 2044 |
| 3 | ORFEI CP | 18 | 424 | LAI RC | 7 | 2029 |
| 4 | COLOMBINI A | 14 | 417 | HUI JHP | 7 | 1680 |
| 5 | LI Y | 14 | 323 | ZHANG SP | 5 | 1170 |
| 6 | WANG Y | 14 | 634 | CHUAH SJ | 2 | 1051 |
| 7 | XU X | 14 | 897 | NOËL D | 8 | 923 |
| 8 | CHEN L | 13 | 903 | JORGENSEN C | 7 | 921 |
| 9 | VIGANÒ M | 13 | 413 | CHEN L | 13 | 903 |
| 10 | XU J | 13 | 332 | XU X | 14 | 897 |
| 11 | DE LUCA P | 12 | 322 | LIANG YJ | 11 | 811 |
| 12 | DUAN L | 11 | 793 | DUAN L | 11 | 793 |
| 13 | LIANG YJ | 11 | 811 | XIA J | 11 | 738 |
| 14 | LIU SY | 11 | 550 | KUANG L | 5 | 726 |
| 15 | TOH WS | 11 | 2116 | NI ZH | 6 | 717 |
| 16 | XIA J | 11 | 738 | RUIZ M | 4 | 654 |
| 17 | ZHANG J | 11 | 206 | TAO SC | 3 | 651 |
| 18 | ZHANG L | 11 | 175 | KANG Y | 5 | 646 |
| 19 | ZHANG X | 11 | 119 | HU S | 4 | 634 |
| 20 | CHEN J | 10 | 281 | LIAO WM | 4 | 634 |

Table S6 Annual publication volume of the top 20 journals

| **Rank** | **Journal** | **Publications** | **Citations (rank)** | **Average citations (rank)** | **Betweenness centrality** |
| --- | --- | --- | --- | --- | --- |
| 1 | INTERNATIONAL JOURNAL OF MOLECULAR SCIENCES | 46 | 725(6) | 15.76(15) | 0.00 |
| 2 | FRONTIERS IN BIOENGINEERING AND BIOTECHNOLOGY | 27 | 727(5) | 26.93(9) | 0.01 |
| 3 | CELLS | 23 | 442(9) | 19.22(13) | 0.04 |
| 4 | JOURNAL OF NANOBIOTECHNOLOGY | 23 | 708(7) | 30.78(6) | 0.00 |
| 5 | STEM CELL RESEARCH & THERAPY | 23 | 2156(1) | 93.74(3) | 0.05 |
| 6 | ARTHRITIS RESEARCH & THERAPY | 17 | 776(4) | 45.65(5) | 0.04 |
| 7 | FRONTIERS IN CELL AND DEVELOPMENTAL BIOLOGY | 15 | 430(10) | 28.67(8) | 0.17 |
| 8 | JOURNAL OF ORTHOPAEDIC SURGERY AND RESEARCH | 12 | 294(12) | 24.50(10) | 0.07 |
| 9 | STEM CELLS INTERNATIONAL | 12 | 263(13) | 21.92(11) | 0.05 |
| 10 | BIOMEDICINES | 11 | 126(16) | 11.45(16) | 0.17 |
| 11 | FRONTIERS IN IMMUNOLOGY | 11 | 234(14) | 21.27(12) | 0.05 |
| 12 | JOURNAL OF ORTHOPAEDIC TRANSLATION | 11 | 321(11) | 29.18(7) | 0.04 |
| 13 | BIOACTIVE MATERIALS | 10 | 542(8) | 54.20(4) | 0.00 |
| 14 | INTERNATIONAL IMMUNOPHARMACOLOGY | 10 | 166(15) | 16.60(14) | 0.16 |
| 15 | PHARMACEUTICS | 9 | 95(17) | 10.56(17) | 0.12 |
| 16 | FRONTIERS IN PHARMACOLOGY | 8 | 72(18) | 9.00(18) | 0.01 |
| 17 | THERANOSTICS | 8 | 1540(3) | 192.50(2) | 0.07 |
| 18 | BIOMATERIALS | 7 | 1985(2) | 283.57(1) | 0.03 |
| 19 | BIOMEDICINE & PHARMACOTHERAPY | 7 | 58(19) | 8.29(19) | 0.05 |
| 20 | HELIYON | 7 | 37(20) | 5.29(20) | 0.01 |
