# Supplementary figures and images for: Bibliometric and LDA analysis of extracellular vesicles in osteoarthritis
Source: Bone Res. 2025 Dec 23;13:105. doi: 10.1038/s41413-025-00484-3 (PMC12722249; doi:10.1038/s41413-025-00484-3)

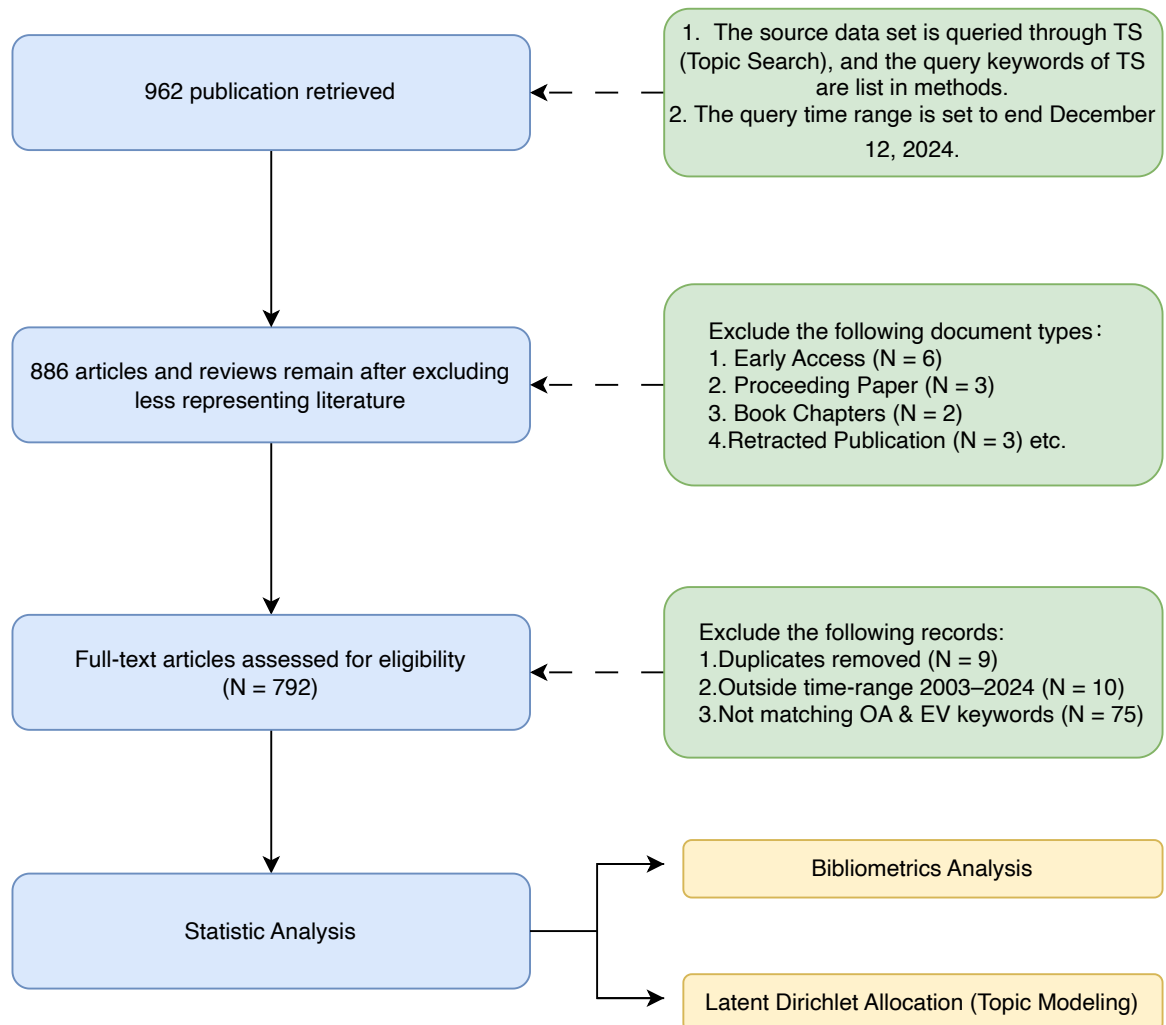

Supplement: Supplementary file 2 — Figure S2 [file 41413_2025_484_MOESM2_ESM.pdf]

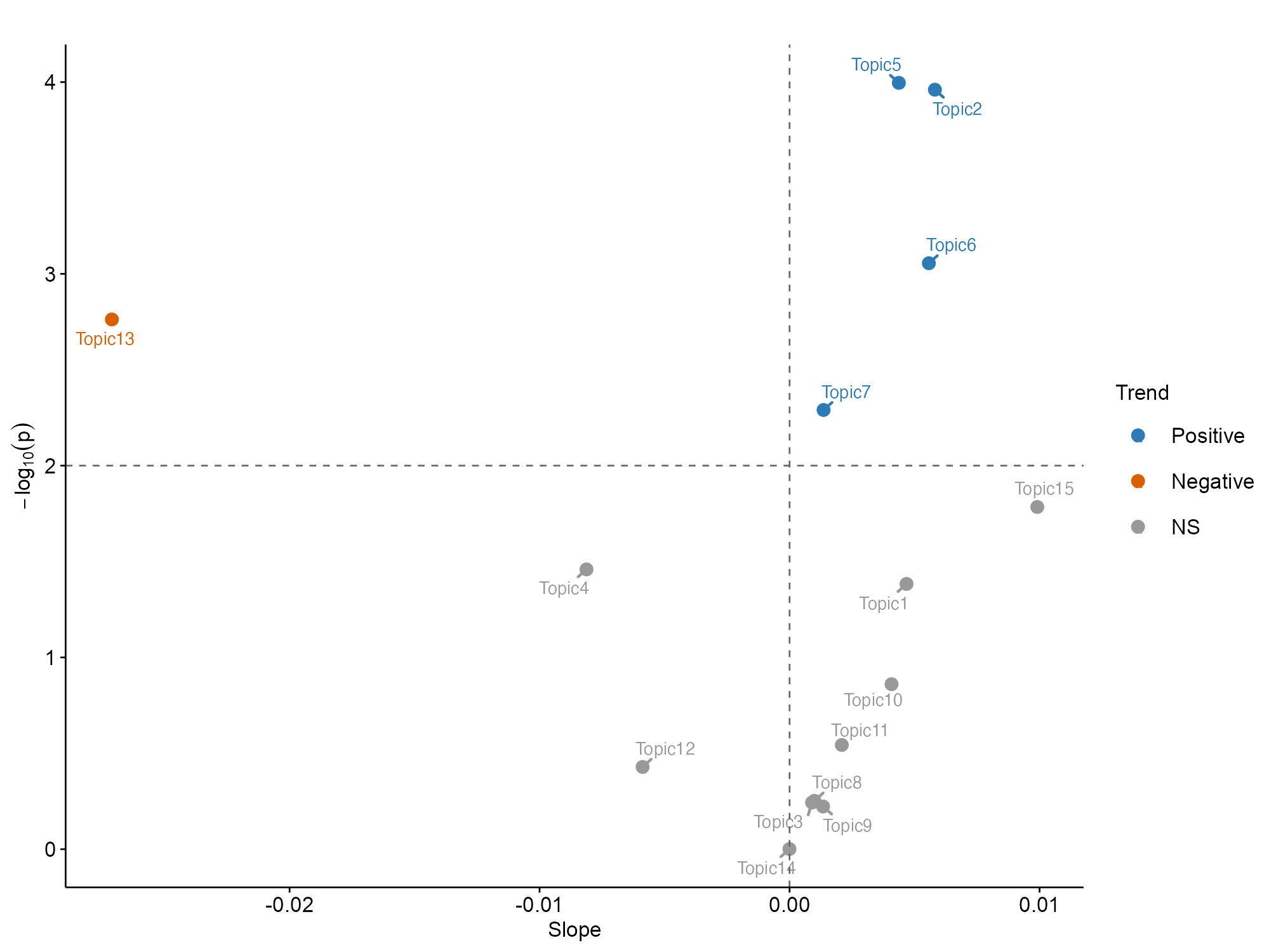

Supplement: Supplementary file 3 — Figure S1 [file 41413_2025_484_MOESM3_ESM.tif]
