# Supplementary material for: Bibliometric and LDA analysis of extracellular vesicles in osteoarthritis
Source: Bone Res. 2025 Dec 23;13:105. doi: 10.1038/s41413-025-00484-3 (PMC12722249; doi:10.1038/s41413-025-00484-3)
Supplement: Supplementary file 4 — Supplementary Figures Legends [file 41413_2025_484_MOESM4_ESM.docx]

Supplementary Files

**Figure S1** Volcano-style plot of topic trend significance. Each point represents one latent topic; the x-axis is the slope of the topic’s annual mean posterior probability over time (from linear regression), with positive values indicating increasing prominence and negative values indicating decline. The y-axis is –log₁₀(P-value) for the null hypothesis that the slope equals zero (higher values denote stronger statistical evidence of change). The vertical dashed line at zero separates rising from falling topics; the horizontal dashed line marks the significance threshold (e.g., P = 0.01, corresponding to –log₁₀(P) = 2). Color coding: blue denotes topics with significantly increasing trends, red denotes significantly decreasing trends (e.g., Topic14), and gray indicates non-significant trends.

**Figure S2** Literature Screening Flowchart
